# Dendritic complexity change in the triple transgenic mouse model of Alzheimer’s disease

**DOI:** 10.7717/peerj.8178

**Published:** 2020-01-09

**Authors:** Yu Zhang, Zhenlong Xiao, Zhijun He, Junyu Chen, Xin Wang, Liang Jiang

**Affiliations:** 1 Shenzhen University, Shenzhen Key Laboratory of Marine Bioresources and Ecology, Brain Disease and Big Data Research Institute, College of Life Sciences and Oceanography, Shenzhen, China; 2 Harbin Institute of Technology (Shenzhen), Department of Mechanical and Automation Engineering, Shenzhen, China

**Keywords:** Alzheimer’s disease, Neuronal morphology analysis, Micro-optical sectioning tomography

## Abstract

Alzheimer’s disease (AD) is an irreversible, neurodegenerative disease that is characterized by memory impairment and executive dysfunction. However, the change of fine structure of neuronal morphology remains unclear in the AD model mouse. In this study, high-resolution mouse brain sectional images were scanned by Micro-Optical Sectioning Tomography (MOST) technology and reconstructed three-dimensionally to obtain the pyramidal neurons. The method of Sholl analysis was performed to analyze the neurons in the brains of 6- and 12-month-old AD mice. The results showed that dendritic complexity was not affected in the entorhinal cortex between 6-month-old mice and 12-month-old mice. The dendritic complexity had increased in the primary motor cortex and CA1 region of hippocampus of 12- month-old mice compared with 6-month-old mice. On the contrary, dendritic complexity in the prefrontal cortex was decreased significantly between 6-month-old and 12-month-old mice. To our knowledge, this is the first study to provide high-resolution brain images of triple transgenic AD mice for statistically analyzing neuronal dendrite complexity by MOST technology to reveal the morphological changes of neurons during AD progression.

## Introduction

Alzheimer’s disease (AD) is a complicated, age-related, degenerative neurological disease (Laureys, Gosseries & Tononi, 2015). Although its pathological features has attracted much attention to study, the attempts to fully control and reverse the process of AD has never been achieved in terms of the ultimate goal (Coman & Nemeş, 2017; Reiman et al., 2016). Current investigation mainly focused on exploring the molecular and genetic mechanisms of AD, such as studying the deposition of *β*-amyloid (A*β*) *protein* and the formation of neurofibrillary tangles (NFT) from tau protein hyperphosphorylation (Blennow et al., 2010). Molecular lesion in the asymptomatic phase of AD is one of the early events that lead to neuronal damage and cognitive decline (symptomatic phase) (Jack Jr et al., 2010). A*β* aggregation and tau hyperphosphorylation are accompanied by the morphological change of neurons, which shows as the change of neuronal shape, volume and complexity. Neuronal morphology determines its connectivity to the other cells and its physiological function in the brain (Brown, Gillette & Ascoli, 2008). The subtle change of morphology can be described by its neuronal complexity including dendritic length, dendritic volume and terminal numbers (Uylings & Van Pelt, 2002), each of which has an impact on the growth and adaptability of the neuron (Wong & Ghosh, 2002). The method of Sholl analysis is thus used to analyze the morphology of neurons (Brown, Gillette & Ascoli, 2008).

Episodic memory and spatial cognition were consider as the function of hippocampus (Scoville & Milner, 1957; Shettleworth, 2003). The hippocampal atrophy is often considered as a direct, validated indicator of AD (Arlt et al., 2013; Convit et al., 1997; De Flores, La Joie & Chételat, 2015). Some studies have suggested that AD is associated with the apoptosis of neurons in the hippocampal tissue (Casas et al., 2004). In the entorhinal cortex, its function includes processing of space and time, and linking to the taste perception (Hwang et al., 2019; Schmidt-Hieber & Häusser, 2013; Tsao et al., 2018). It was found that volume of entorhinal cortex had significantly altered during AD (Khan et al., 2014). Only when relatively large neuronal loss occurs (when 35% of the entorhinal cortex is involved), the patients begin to develop the corresponding clinical symptoms also known as a very mild AD stage (Price et al., 2001). The prefrontal cortex gray matter’s degeneration has been found to be associated with AD (Salat, Kaye & Janowsky, 2001). The primary motor cortex, which controls the execution of movement, has also been reported to be involved in AD pathological process (Suva et al., 1999). The morphological changes of neurons in those regions of AD brain have never been reported previously.

In order to reveal the change of neurons during the pathological process of AD, high resolution images must be obtained for statistical analysis of the subtle changes of neurons. Traditional neuron imaging technology is not precise enough to provide fine structural observation of a wide range of neurons. In order to achieve this purpose, we introduced the newly developed method of micro-optical sectioning tomography (MOST) (Li et al., 2010) to investigate the morphological change of neurons in different brain regions of the triple transgenic AD model mouse (3 × Tg-AD) at the ages of 6 and 12 months (Oddo et al., 2003).

## Materials and Methods

### Animals

The triple transgenic AD model mice (3 × Tg-AD) carrying human gene mutants APPswe, PS1M146V, and tauP301L were purchased from the Jackson laboratory (Bar Harbor, ME, USA). Six male 3 ×Tg AD mice were selected for the experiments of MOST technology, among them three were 6-month-old (abbreviated as AD06) and another three were 12-month-old (AD12).

All the animals were housed in an environment with a temperature of 22  ± 1 °C, relative humidity of 50  ± 1% and a light/dark cycle of 12/12 hr. Additionally, all animal studies (including the mice euthanasia procedure) were done in compliance with the regulations and guidelines of Shenzhen University Institutional Animal Care Center, Experimental Animal Ethics Committee of Shenzhen University Medical Department and the AAALAC and the IACUC guidelines (animal experiment proof certificate number: SYXK2014-0140).

### Whole brain Golgi staining

In consideration of the morphological observation of neuronal dendrites, Golgi staining was chosen as the staining method. Mice were euthanized and brains were removed and placed in the Golgi-cox (Zhang et al., 2011) solution for fixation and impregnation, and stored at room temperature for 2 months. Golgi-Cox solution consists of the following ingredients: 1 g mercuric chloride, 1 g potassium dichromate, and 0.8 g potassium chromate with 80 g ddH_2_O. Then, the solution of 1% lithium hydroxide was used to immerse brains for 24 h. The rinsed brains were sequentially immersed in 50%, 70%, 85%, 95%, 100% alcohol, 100% alcohol-acetone (1:1), and 100% acetone (2x) for dehydration. After dehydration, the brain was infiltrated by 50%, 75%, and 100% Spurr resin (2x) and was maintained at 60 °C for 36 h polymerization.

### Brain treatment, data collection and preliminary handling

MOST systems was used to collect data from the mouse brain with a voxel size of 0.35 µm × 0.35 µm × 1 µm. The MOST system consists of a microtome, an optical microscope and an image recorder that simultaneously slice and image (40×, numerical aperture 0.8) the sample. At work, the microtome cuts the sample into strips having a width of about 450 µm. Once separated from the sample block, the ribbon is imaged immediately. An optical microscope is a reflective bright-field microscope in which the illumination beam is perpendicular to the rake face of the blade and coincides with the imaging beam (Li et al., 2010).

Raw data was pretreated with MATLAB (Ding et al., 2013). The most original data were image tiles, which was spliced to obtain a complete image. Periodic noise of faults was corrected by mean projection curve and calibrated by nonuniform illumination. At the same time, the strength of the effective data area of each fault was adjusted to the uniform intensity to overcome different intensity of the faults in the integrated strips.

### Three-dimensional reconstruction

According to the anatomical map of mouse brain (Paxinos & Franklin, 2008), the position of four regions were located in the following stereotaxic coordinates: the prefrontal cortex (Bregma, 3.08∼1.54 mm), the primary motor cortex (Bregma, 2.34∼-1.22 mm), the entorhinal cortex (Bregma, -4.04∼-5.02 mm) and the hippocampus CA1 (Bregma, -1.22∼-3.88 mm). A block size of 600 ×600 × 600 µm^3^ was chosen in the brain for statistical analysis of the parameters of neuronal morphology. The Amira software (version 5.4) that provides an interactive interface was used to reconstruct each sub-block and to correct the images and pyramidal cells in three dimensions for visualizing the neurons inside.

### Estimation of neuronal density

To count neurons within the brain areas, the description and boundaries of brain regions was defined by a mouse brain atlas (Paxinos & Franklin, 2008). No attempt to identify target regions subdivisions was made. Following a systematically random scheme and based on stereological unbiased techniques, five blocks (300 × 300 × 300 µm^3^ for each block) were selected, taken at equally spaced intervals (3 mm) along the entire length of the target brain region on each brain region (Gómez-Isla et al., 1996).

Considering the complexity of Golgi stained image, manual labeling cell centroids method was selected. Image stacks are aligned in Amira 5.4.1 (Visage Imaging). We manually label markers at the center for each cell with vision perception (Meng et al., 2014; Wu et al., 2014). All large cells (most likely neurons), but not small cells (which could be small neurons or glia), were counted.

### Statistical analysis

A series of statistical methods for analyzing neuronal data were written through MATLAB in this paper(Cuntz, Borst & Häusser, 2011). Those neuronal data include dendritic length, number of terminal branches, number of Sholl intersections, and spatial volume of neurons in different brain regions of different mouse groups (Ascoli et al., 2008; Caserta et al., 1995; Ristanović, Milošević & Štulić, 2006).

Dendritic length referred to the sum of the lengths of all dendritic branches of neurons (µm). The number of terminal dendrites referred to the number of branches of the last stage of neuronal dendrites (number). The spatial volume of a neuron was the smallest cubic volume that could accommodate the entire neuron (µm^3^). The number of Sholl analysis intersections is counted from the method of Sholl analysis that is commonly used to analyze the morphology of dendrites (Gensel et al., 2010). Based on the Sholl analysis, the soma of a neuron was at the center and the circle was drawn with a radius of 30 µm each time. These concentric circles reflected the distance of the dendrites to the soma. The number of intersections between the concentric circles and the dendrites reflected the number of dendrites at that distance. For statistical analysis of those data (including dendritic length, terminal dendrites number, the number of neurons and the number of intersections in Sholl analysis), *t*-test was used to analyze the differences among groups. Confidence level was set to 0.05 (*p*-value) and all the results are presented as the mean  ± SEM.

## Results

### Dendrite tracking in the brain of 3 × Tg-AD mouse

**Figure 1:**
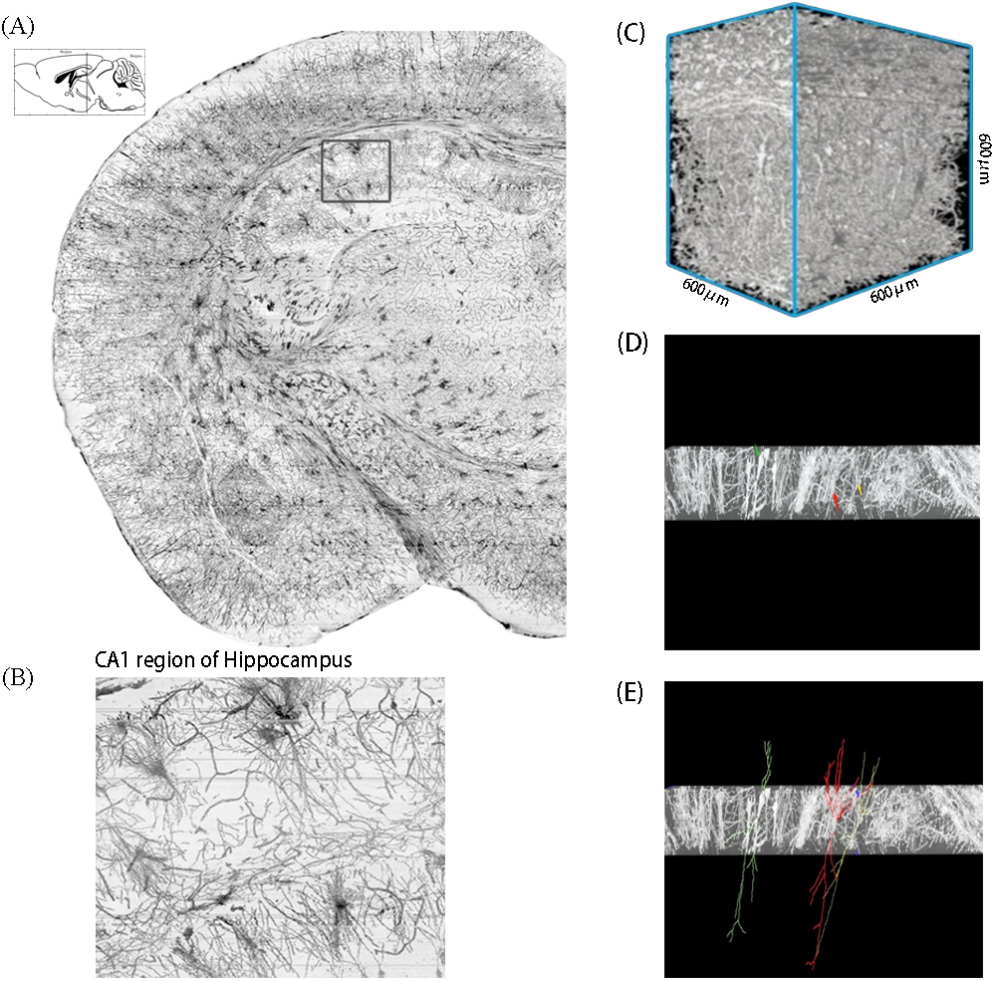
Procedures for the neuron tracking process. (A) A coronal projection image, showing the left brain section of an AD model mouse, was located between Bregma-1.22 to -4.04 that contained a CA1 region of hippocampus. (B) An enlarged view of the cropped image. (C) The block, containing a series of neuronal pictures, was reconstructed in the Amira. (D) By changing the block thickness and direction, the neuron was clearly displayed. The soma was detected at the first time. Different color (green, red and yellow) arrows indicated the neurons that would be tracked. (E) Dendrites was tracked in different neurons.

Using the interactive neuron tracking function of Amira software, pyramidal neurons were selected according to morphology criteria (Sah et al., 2003) and tracked in the four regions of the AD mouse brain. As shown in Fig. 1A, target brain region was detected and confirmed in the projection of serial coronal sections. Neuronal details were showed in the projection photo which contains the information of a brain slice with 50 µm thickness. The photo of target region, the CA1 region of hippocampus, was cropped to a set of 600 × 600 × 600 µm^3^ blocks. Soma and the dendrites were shown clearly (Fig. 1B) in this block, which could be reconstructed by Amira and revealed in three-dimension (Fig. 1C). By changing the layer thickness and direction, the nerve fibers can be clearly displayed. During the tracking process, the soma of pyramidal neuron was located firstly (Fig. 1D), and the neuronal dendrites were extended from the soma (Fig. 1E).

**Figure 2:**
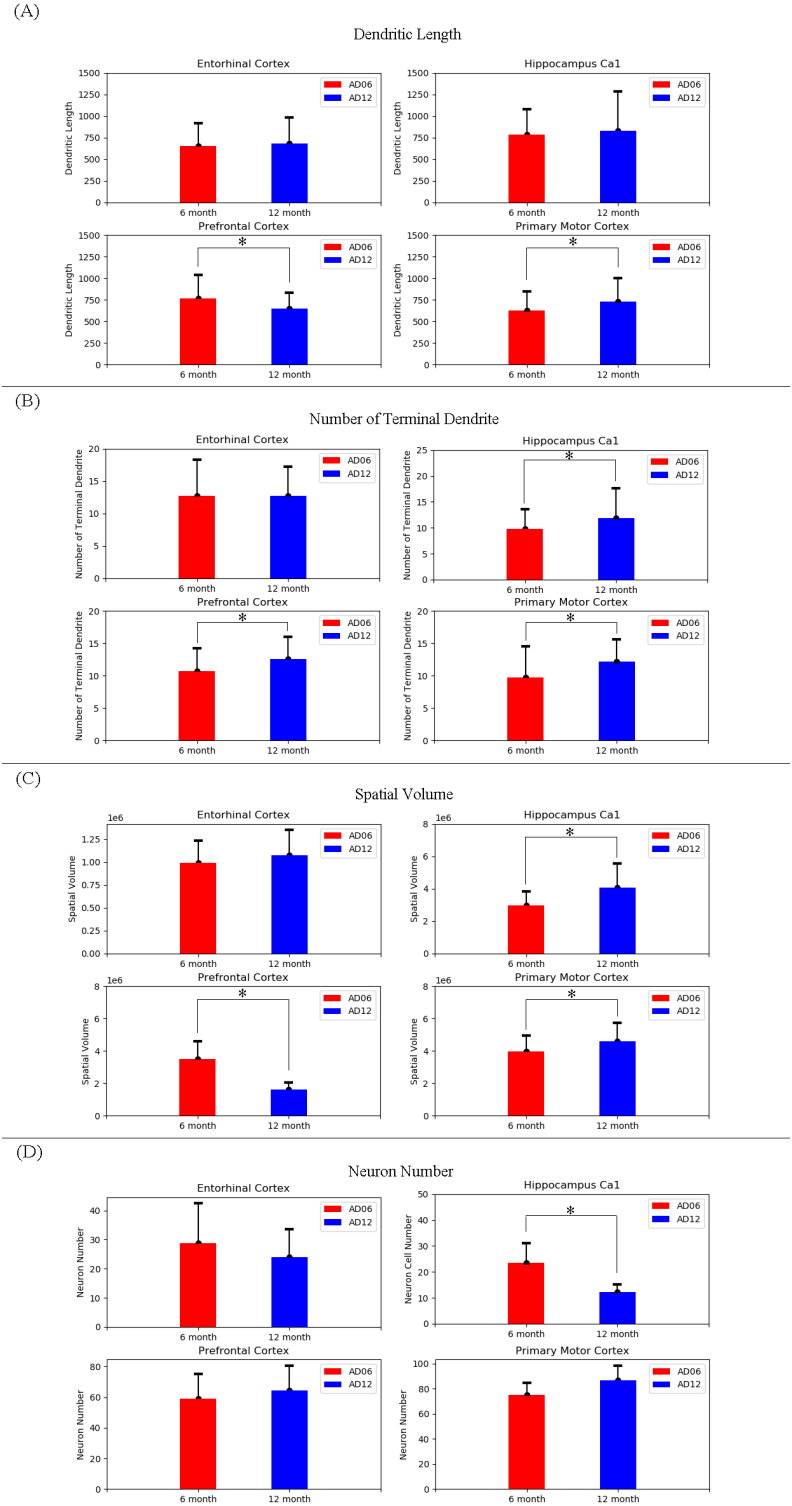
Dendritic parameters and neuron number in the four brain regions of AD model mice at the 6 and 12 month-old. (A) The analysis of neuron number and neuron morphology in the entorhinal cortex; (B) The analysis of neuron number and neuron morphology in the prefrontal cortex; (C) The analysis of neuron number and neuron morphology in the primary motor cortex; (D) The analysis of neuron number and neuron morphology in the CA1 region of hippocampus.

### Dendritic complexity and soma counting in different brain regions

In order to investigate the overall change of neuron morphology, dendritic complexity in terms of dendritic length, the number of terminal dendrites and spatial volume were measured, followed by the number of neurons. As shown in Fig. 2, in the entorhinal cortex region, no significant change was found in the number of neurons (28.75 ± 6.9 for AD06, 24 ± 9.6 for AD12), neuronal dendritic length (650.6 ± 268.72 µm for AD06, 682.40 ± 300 µm for AD12), the number of terminal dendrites (12.68 ± 5.6 for AD06, 12.7 ± 4.53 for AD12) and the spatial volume of neuron (0.98 × 10^6^ ± 0.24 × 10^6^ µm3 for AD06, 1.07 × 10^6^ ± 0.27 × 10^6^ µm3 for AD12).

In the prefrontal cortex region, a significant decrease was measured in the dendritic length of AD12 (645.58 ± 187.9 µm, *n* = 60, *p* = 0.007) compared to the values of the AD06 (762.47 ± 276.3 µm) and in the spatial volume of neuron of AD12 (1.62 ×10^6^ ± 0.41 × 10^6^ µm^3^, *n* = 60, *p* = 0.001) compared to the values of the AD06 (3.51 × 10^6^ ± 1.08 × 10^6^ µm^3^). Additionally, a significant increase was found in the number of terminal dendrites of AD12 (12.53 ± 3.45, *n* = 60, *p* = 0.004) compared to the values of the AD06 (10.7 ± 3.5). No significant change was found in the number of neurons (59.1 ± 15.9 for AD06, 64.4 ± 16.3 for AD12).

In the primary motor cortex region, there was a significant increase in the dendritic length of AD12 (730.61 ± 268.59 µm, *n* = 60, *p* = 0.02) compared to the values of the AD06 (628.19 ± 215.8 µm) and in the number of terminal dendrites of AD12 (12.17 ± 3.39, *n* = 68, *p* = 0.001) compared to the values of the AD06 (9.72 ± 4.76) and in the spatial volume of AD12 (4.5 × 10^6^ ± 1.1 ×10^6^ µm^3^, *n* = 51, *p* = 0.003) compared to the values of the AD06 (3.9 × 10^6^ ± 0.96 × 10^6^ µm^3^). No significant change was found in the number of neurons (74.8 ± 10 for AD06, 86.7 ± 11.7 for AD12).

In the CA1 region of hippocampus, no significant change was measured in neuronal dendritic length in each group (785.4 ± 295.22 µm for AD06, 829.86 ± 402.2 µm for AD12). However, the number of neurons in this region reduced significantly in AD12 group (12.2 ± 2.8, *n* = 5, *p* = 0.01) compared to AD06 group (23.4 ± 7.7). As compensation, a significant increase was measured in the number of terminal dendrites of AD12 (11.85 ± 5.7, *n* = 60, *p* = 0.02) compared to the values of the AD06 (9.78 ± 3.7) and in the spatial volume of neuron of AD12 (4.08 × 10^6^ ± 1.48 × 10^6^ µm^3^, *n* = 49, *p* = 0.001) compared to the values of the AD06 (2.98 × 10^6^ ± 0.84 × 10^6^ µm^3^).

### Distribution of neuron dendritic density in different brain regions

**Figure 3:**
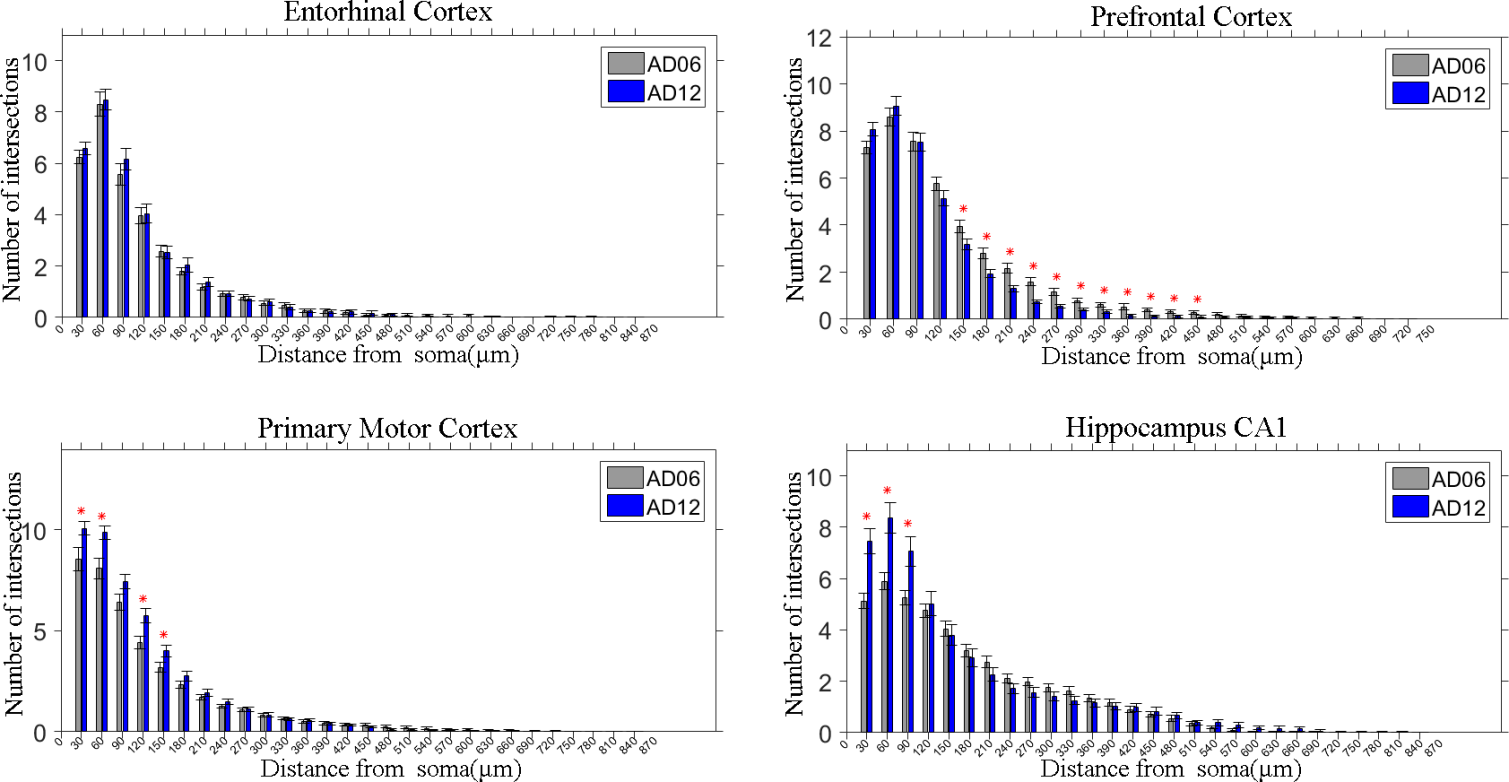
Sholl analysis of neuronal dendrites in four brain regions of AD model mouse. Sholl analysis results shown the number of intersections between the dendrites and the circles that centered at the soma with different distances.

Three-dimensional Sholl analysis was used to indicate the dendritic density in a radial direction. As shown in Fig. 3, in the entorhinal cortex region, no significant change of intersections was measured between the AD06 and AD12 groups. In the prefrontal cortex region, the AD06 group had significantly more intersections in the 150–450 µm ranges than those of the AD12 group (*p* = 0.001), indicating that the number of dendrites in the AD12 group decreased in a radial direction. In the primary motor cortex region, the AD12 group had significantly more intersections in the 30–60 µm and 120–150 µm ranges than those of the AD06 (*p* = 0.03), indicating that the increase of dendrites in the AD12 group were around the soma. In the CA1 region of hippocampus, the AD12 group had significantly more intersections in the 30–90 µm ranges than those of the AD06 group (*p* = 0.001), indicating that the number of dendrites in the AD12 group increased in the region closed to the soma.

## Discussion

In this study, triple transgenic AD mice 3 × Tg-AD at the ages of 6 and 12 months were used for high-resolution imaging by the MOST technology and for reconstruction of the neuron morphology three-dimensionally through data mining. Neuronal morphology was analyzed and evaluated by dendritic density in a radial direction and neuronal complexity in terms of dendritic length, the number of terminal dendrites and the spatial volume in four brain regions. Meanwhile, the number of neurons in the same four regions was also counted. Results in this paper showed that neither dendritic density and complexity nor the number of neurons altered significantly in the entorhinal cortex between AD06 and AD12 groups. In the prefrontal cortex, the number of neurons remained unchanged in AD12 mice compared with AD06 mice, but the dendritic length and the spatial volume of a neuron decreased significantly and the number of terminal dendrites increased significantly. Meanwhile dendritic density also decreased in a radial direction in the AD12 group. Those indicated that the damage of neurons occurred in this brain region at the age of 12 months and as compensation the dendritic terminals increased. In the primary motor cortex, dendritic complexity increased significantly in AD12 and the number of neurons did not change significantly. However, dendritic density increased mainly around the soma in AD12 group, indicating that the pathological process did not significantly affect the neurons with shorter dendrites in the primary motor cortex. In the hippocampal CA1 region, the number of neurons decreased significantly in AD12 mice and dendritic density increased in a region very close to the soma. As a compensation, neuronal complexity in terms of the number of terminals and the spatial volume increased significantly.

Dendritic length, number of terminal dendrites and spatial volume are important indicators for describing neuronal morphology (Ascoli et al., 2008). The spatial volume of a neuron corresponds to the invading spatial region of the neuron in the brain tissue and the potential connectivity in the brain region. For axons, this can lead to increased signal bifurcation (a signal is sent to many cells) (Brown, Gillette & Ascoli, 2008). Dendritic length is minimized by increasing length for axons over dendrites when divergence is higher than convergence. When convergence is greater than divergence, dendrites have relatively greater length (Chklovskii, 2000).

Amyloid-*β* (A*β*) is considered as one of the main marker of AD. In 3 × Tg AD mice, both of these markers appeared relatively early. A*β* deposition was found in the new cortex at the age of 4 months (Yan-qiu, 2015), while A*β* deposition appears in the frontal cortex and hippocampal CA1 at the age of seven months (Oddo et al., 2003). A*β* appears in most of the pyramidal neurons by the age of 6 months (Mastrangelo & Bowers, 2008). In addition, with the advent of AD markers, a decrease in the number of neurons occurs in brain regions. As disorder severity deepens, the number of neurons in the hippocampal CA1 and CA2 decreases (Zarow et al., 2005). Neuron number reduction is one of the AD process markers (Serrano-Pozo et al., 2011). The activity of AD mice behavior did not decreased, even increased as the accumulation of A*β* in the brain. 3 × Tg AD mice had increased motor function on Rotarod (Stover et al., 2015). Moreover, The behavior of the double transgenic APPswe/PS1dE9 6-month-old mice was the same as that of the normal mice, but changed at the age of 12-months (Lin, Zhi-jun & Min, 2013).

The current theory cannot explain why cognitive decline lags behind the appearance of AD molecular markers and nerve damage. Another study suggests that nerve cells have the ability to respond to cell-induced damage through morphological transition (Gastinger et al., 2008). This further implies that there might be a compensation mechanism in the AD progress. By changing the morphology of neurons, the brain compensates for the defects of the neural network caused by factors such as apoptosis.

Our results shows that the change of dendrite complexity exists differently in different brain region. The dendritic complexity was not affected in the entorhinal cortex between 6-month-old mice and 12-month-old mice. The dendritic complexity had increased in the primary motor cortex and CA1 region of hippocampus of 12-month-old mice compared with 6-month-old mice. On the contrary, dendritic complexity in the prefrontal cortex was decreased significantly. A compensation mechanism may exists in CA1 region of hippocampus with neuron number decreased and complexity increased. Additionally, it also may exists in the prefrontal cortex in terms of dendritic length decreased and spatial volume decreased and terminal dendrite number increased. In the APP/PS1 model, abnormalities in dendritic morphology can lead to hyperexcitability in neurons (Šišková et al., 2014). Analysis of the active state of neurons found that the introduction of the mutant A*β* triggered neuronal overactivity (Busche et al., 2008). Dendritic geometry and neuron function are inseparably linked, defining the dendritic integration of synaptic signals, their propagation, and their capability to evoke action potential output (Magee, 2000; Poirazi & Mel, 2001; Spruston, 2008). The complex treelike architecture of the dendrites receive the vast majority of the cell’s synaptic input, and act as the primary substrate for neuronal information processing (Häusser & Mel, 2003). When the neurons are in this abnormal excitability, the change of dendritic morphology needs further exploration. Next, we will analysis the morphological changes between the AD model mice and wild type mice.

## Conclusion

Alzheimer’s disease is a neurodegenerative disease that is irreversible with a complex pathogenesis. Early diagnosis and prevention of AD is a difficult and challenging work. Advances in cross-disciplinary research in different disciplines can provide diverse perspectives for Alzheimer’s basic clinical research, especially using three-dimension imaging. The MOST system provides a powerful way to observe the neuron morphology in high resolution. With the application of MOST system and image analysis, we found that the number of neurons did not change significantly in three cortex regions. However, neuronal morphologies in terms of dendritic density and complexity changed differently in three cortex regions between the A06 and A12. Neurons in the entorhinal cortex was not affected by the pathological progression of AD, while neurons with shorter dendrites increased their dendritic complexity in the primary motor cortex of 12-month-old mice. On the contrary, neurons in the prefrontal cortex was damaged significantly, especially for these neurons with long dendrites in the mice of 12-month-old. In the hippocampal CA1 region, the reduced number of neurons was compensated with increased dendritic complexity and density close to the soma in the 12-month-old mice. The results in this study help to understand the relation between neuronal morphology and the pathology of AD.

##  Supplemental Information

10.7717/peerj.8178/supp-1 Supplemental Information 1 Analysis code of neuron data after neuron tracking The Statistics file is used to analyze neuron data to obtain indicator information of neurons. The Main file in the Statistics file provides the indicators calculation entry. Sholl_statics_main.m provides sholl analysis results. Neuron_cluster.m and Dendrite_Analysis.m provide neuron indicators. The api of the TREE file is used in the file, which we mentioned in the Methods. The appfile is mainly used for image processing of MOST and provides a part of the api for Statistics. Neuron_result_to_photo.py represents the results of various indicators as images.

10.7717/peerj.8178/supp-2 Supplemental Information 2 Raw data from neuron tracking in Amira Raw data contains 675 neuron data. 468 belonged to Alzheimer disease(AD) mice. 207 were from wild type mice. In the article, we only analyzed for AD mice. Wild type mice neuron data are part of our additional reference. The data relates to 4 brain regions (primary motor cortex, prefrontal cortex, hippocampus CA1, entorhinal cortex). The information of the neurons in the file name of the neuron file includes the mouse category, the brain region abbreviation, the left and right brain hemisphere, the layer position, and the neuron number.

## Additional Information and Declarations

### Data Availability

The following information was supplied regarding data availability: The neuron data is available in the Supplemental File and at figshare: chace (2019): Neuron_data. figshare. Dataset. 10.6084/m9.figshare.8020400.v1. The neuron analysis code in the Supplemental File and at figshare: chace (2019): Analysis_code. figshare. Software. 10.6084/m9.figshare.8020580.v4.
